# Poly[diaqua-μ_3_-4-nitro­phthalato-copper(II)]

**DOI:** 10.1107/S1600536810049792

**Published:** 2010-12-04

**Authors:** Ming-Lin Guo

**Affiliations:** a School of Environment and Chemical Engineering and Key Laboratory of Hollow Fiber Membrane Materials and Membrane Processes, Tianjin Polytechnic University, Tianjin 300160, People’s Republic of China

## Abstract

In the title complex, [Cu(C_8_H_3_NO_6_)(H_2_O)_2_]_*n*_, the two carboxyl­ate groups of the 4-nitro­phthalate dianion ligands have monodentate and 1,3-bridging bonding modes, respectively. The Cu atom shows an approximate square-pyramidal coordination as it is bonded to O atoms from the carboxyl­ate groups of three 4-nitro­phthalate ligands and two O atoms of the non-equivalent coordinated water mol­ecules. Other Cu atoms in the coordination polymer are connected into a two-dimensional layer in the *ab* plane. The layers are aggregated to a three-dimensional structure through inter­layer hydrogen bonding involving an O atom of a nitro group. The whole three-dimensional structure is further maintained and stabilized by intra­layer hydrogen bonds between the O atoms of the carboxyl­ate groups and the coordinated water mol­ecules.

## Related literature

For τ value calculations in a square-pyramidal environment, see: Addison *et al.* (1984 ▶). For related structures, see: Baca *et al.* (2003 ▶, 2004 ▶); Biagini Cingi *et al.* (1978 ▶); Fu *et al.* (2006 ▶); Guo & Guo (2007 ▶); Ma *et al.* (2004 ▶); Wang *et al.* (2009 ▶); Yang *et al.* (2003 ▶). For hydrogen bonds, see Bernstein *et al.* (1995 ▶); Brown (1976 ▶). For a comparison of Cu—O distances, see: Pasan *et al.* (2007 ▶).
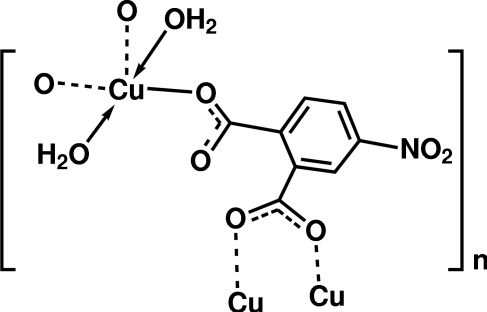


## Experimental

### 

#### Crystal data

[Cu(C_8_H_3_NO_6_)(H_2_O)_2_]
*M* *_r_* = 308.69
Orthorhombic, 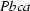

*a* = 14.208 (3) Å
*b* = 6.5159 (13) Å
*c* = 21.722 (4) Å
*V* = 2011.0 (7) Å^3^
*Z* = 8
Mo *K*α radiation
μ = 2.21 mm^−1^
*T* = 133 K
0.14 × 0.06 × 0.04 mm

#### Data collection

Rigaku Saturn diffractometer
Absorption correction: multi-scan (*CrystalClear*; Rigaku/MSC, 2005 ▶) *T*_min_ = 0.850, *T*_max_ = 0.917
11779 measured reflections
1974 independent reflections
1609 reflections with *I* > 2σ(*I*)
*R*_int_ = 0.079

#### Refinement

*R*[*F* ^2^ > 2σ(*F* ^2^)] = 0.048
*wR*(*F* ^2^) = 0.144
*S* = 1.07
1974 reflections
163 parameters
H-atom parameters constrained
Δρ_max_ = 0.79 e Å^−3^
Δρ_min_ = −0.66 e Å^−3^

### 

Data collection: *CrystalClear* (Rigaku/MSC, 2005 ▶); cell refinement: *CrystalClear*; data reduction: *CrystalClear*; program(s) used to solve structure: *SHELXS97* (Sheldrick, 2008 ▶); program(s) used to refine structure: *SHELXL97* (Sheldrick, 2008 ▶); molecular graphics: *SHELXTL* (Sheldrick, 2008 ▶); software used to prepare material for publication: *SHELXTL* .

## Supplementary Material

Crystal structure: contains datablocks I, global. DOI: 10.1107/S1600536810049792/sj5060sup1.cif

Structure factors: contains datablocks I. DOI: 10.1107/S1600536810049792/sj5060Isup2.hkl

Additional supplementary materials:  crystallographic information; 3D view; checkCIF report

## Figures and Tables

**Table 1:** Selected bond lengths (Å)

Cu1—O4^i^	1.917 (3)
Cu1—O1	1.945 (3)
Cu1—O8	1.991 (3)
Cu1—O7	1.991 (4)
Cu1—O3^ii^	2.263 (3)

Symmetry codes: (i) 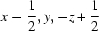; (ii) 
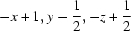
.

**Table 2:** Hydrogen-bond geometry (Å, °)

*D*—H⋯*A*	*D*—H	H⋯*A*	*D*⋯*A*	*D*—H⋯*A*
O7—H7*A*⋯O2^iii^	0.85	2.19	2.862 (5)	136
O7—H7*A*⋯O3^i^	0.85	2.30	2.858 (5)	123
O7—H7*B*⋯O6^iv^	0.85	2.15	2.959 (6)	158
O8—H8*A*⋯O1^v^	0.85	1.98	2.787 (4)	160
O8—H8*B*⋯O2^ii^	0.85	1.85	2.692 (5)	170

Symmetry codes: (i) 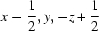; (ii) 
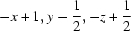
; (iii) 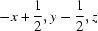; (iv) 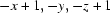; (v) 
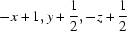
.

**Fig. 1.**
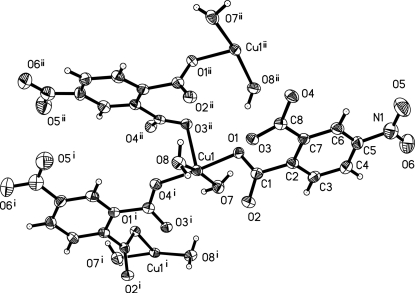
A view of the structure of (I), showing the atom-numbering scheme and coordination polyhedra for the Cu atoms; displacement ellipsoids were drawn at the 30% probability level [Symmetry codes: (i) x - 1/2, y, -z + 1/2; (ii) -x + 1, y - 1/2, -z + 1/2].

**Fig. 2.**
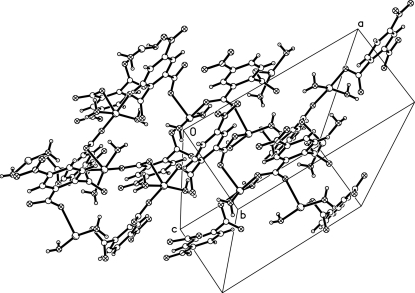
A view of the packing of (I), viewed down the c axis, showing the two-dimensional layer in the ab plane.

**Fig. 3.**
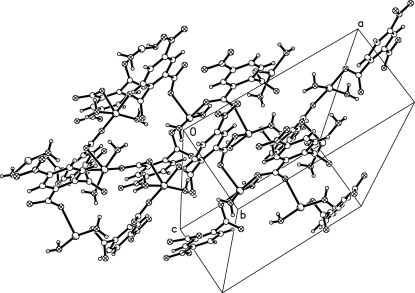
Packing diagram for (I), viewed down the b axis, showing the hydrogen bonding interactions as dashed lines.

## supplementary crystallographic information

### Comment

Aromatic dicarboxylate ligands such as phthalate (phth) and substituted phthalatehave been used in the construction of polymeric metal complexes because they can act as a bis-monodentate, bis-bidentate and combined modes of coordination to form short bridges *via* one carboxylato end or long bridges *via* the benzene ring and lead to a great variety of structures (Biagini Cingi *et al.*, 1978; Guo and Guo, 2007; Wang *et al.*, 2009; Ma *et al.*, 2004; Baca *et al.*, 2003, 2004; Yang *et al.*, 2003; Fu *et al.*, 2006). We have used the 4-nitrophthalate dianion as a ligand, and have obtained the title novel five-coordinate 4-nitrophthalate-copper complex, (I), which forms a three-dimensional supramolecular network through  O—H···O hydrogen bonding.

The asymmetric unit in the structure of (I) comprises one Cu atom, one complete 4-nitrophthate dianion and two non-equivalent water molecules, and is shown in Fig. 1 in a symmetry-expanded view, which displays the full coordination of the Cu atom. Selected geometric parameters are given in Table 1.

The Cu atom exhibits an approximate square pyramidal environment (the τ value being 0.171, Addison *et al.*, 1984), with atoms O1, O4^i^ (see Fig. 1 for symmetry codes) of two non-equivalent 4-nitrophthalate dianions and O7 and O8 atoms of coordinated water molecules in a planar arrangement, with the mean Cu–O(eq) bond distance being 1.961 (3) Å, which is comparable to that reported for poly[(µ_3_-methylmalonato-*O*,*O*',*O*'',*O*''')-aqua-\ copper(II)] (Pasan, *et al.*, 2007). The apical position is occupied by O3^ii^ atom [Cu1–O3^ii^ = 2.263 (3) Å]. The Cu atom is shifted by 0.0889 (5) A° toward the apical position. There is an additional weak Cu–O2 contact in (I), with a Cu···O distance of 2.821 (3) Å.

In the present structure, monodentate, bidentate 1,3-bridging bonding and 1,6-bridging bonding modes *via* the benzene ring are present (Fig. 2). The O1 atom binds in a monodentate fashion, while the O3 and O4 atoms display both monodentate and bidentate 1,3-bridge bonding to link two Cu atoms. The O1 and O3 (or O4) atoms adopt a 1,6-bridging bonding mode *via* the benzene ring to connect with two other Cu atoms.

The Cu atoms are further interconnected by three O atoms from three 4-nitrophthalate dianions into a two-dimensional layer in the *ab* plane. The mean planes of the carboxylate groups of O1/C1/O2 and the benzene ring make a dihedral angle of 72.4 (5)°, and the value of a dihedral angle for the carboxylate groups of O3/C8/O4 is 14.5 (5)°; the two C—O bond distances (O1—C1 and O2—C1) of the monodentate carboxylate group are 1.278 (5) and 1.241 (5) Å, respectively, and the two C—O bond distances (O3—C4 and O4—C4) of the 1,3-bridging bonding carboxylate group are 1.253 (5) and 1.266 (6) Å, respectively. These indicate that the mesomeric effect for the 1,3-bridging bonding carboxylate group is somewhat greater than that of the monodentate carboxylate group.

The two water molecules within the coordination sphere of the Cu atom, and the nitro group (O5/N1/O6) in the present structure engage in distinct hydrogen bonding interactions (see Table 2). Within each layer, the non-coordinated O2 atom is involved in forming  strong O8—H8B···O2^ii^ (Brown, 1976) and weak O7—H7A···O2^v^ hydrogen bonds. These play an important role in the propagation of the two-dimensional layer structure, due to the formation of different hydrogen bonded ring graph set motifs (Bernstein *et al.*, 1995), such as an S(8), and two 10-membered *R*_2_^2^(10) motifs (Fig.3). The neighbouring layers are linked together *via* weak O7—H7B···O6^vi^ hydrogen bonding interactions. These also result in the aryl rings of the 4-nitrophthalato ligands stacking weakly in an offset fashion along the c direction with centroid to centroid distances in the range 4.55 (4)Å - 4.97 (2)%A. Thus, the three-dimensional connectivity of the structure is achieved.

### Experimental

Copper(II) oxide (0.32 g 4 mmol) was added to a stirred solution of 4-nitrophthalic acid (0.53 g, 2.5 mmol) in boiling water (20.0 ml) over a period of 40 min. After filtration, slow evaporation over a period of a week at room temperature provided green needle-like crystals of (I).

### Refinement

All water H atoms were found in difference Fourier maps. However, during refinement, they were fixed at O–H distances of 0.85 Å, with *U*_iso_(H)=1.2 *U*_eq_(O). The H atoms of C–H groups were treated as riding, with C–H = 0.93 Å and *U*_iso_ (H) = 1.2 *U*_eq_(C).

### Crystal data

**Table:**

[Cu(C_8_H_3_NO_6_)(H_2_O)_2_]	*F*(000) = 1240
*M**_r_* = 308.69	*D*_x_ = 2.039 Mg m^−^^3^
Orthorhombic, *P**b**c**a*	Mo *K*α radiation, λ = 0.71073 Å
Hall symbol: -P 2ac 2ab	Cell parameters from 3445 reflections
*a* = 14.208 (3) Å	θ = 2.4–26.0°
*b* = 6.5159 (13) Å	µ = 2.21 mm^−^^1^
*c* = 21.722 (4) Å	*T* = 133 K
*V* = 2011.0 (7) Å^3^	Needle, green
*Z* = 8	0.14 × 0.06 × 0.04 mm

### Data collection

**Table:**

Rigaku Saturn diffractometer	1974 independent reflections
Radiation source: rotating anode	1609 reflections with *I* > 2σ(*I*)
confocal	*R*_int_ = 0.079
Detector resolution: 26.033 pixels mm^-1^	θ_max_ = 26.1°, θ_min_ = 2.4°
ω scans	*h* = −17→15
Absorption correction: multi-scan (*CrystalClear*; Rigaku/MSC, 2005)	*k* = −6→8
*T*_min_ = 0.850, *T*_max_ = 0.917	*l* = −26→26
11779 measured reflections	

### Refinement

**Table:**

Refinement on *F*^2^	Primary atom site location: structure-invariant direct methods
Least-squares matrix: full	Secondary atom site location: difference Fourier map
*R*[*F*^2^ > 2σ(*F*^2^)] = 0.048	Hydrogen site location: inferred from neighbouring sites
*wR*(*F*^2^) = 0.144	H-atom parameters constrained
*S* = 1.07	*w* = 1/[σ^2^(*F*_o_^2^) + (0.0713*P*)^2^ + 4.9336*P*] where *P* = (*F*_o_^2^ + 2*F*_c_^2^)/3
1974 reflections	(Δ/σ)_max_ < 0.001
163 parameters	Δρ_max_ = 0.79 e Å^−^^3^
0 restraints	Δρ_min_ = −0.66 e Å^−^^3^

### Special details

**Table:**

Geometry. All e.s.d.'s (except the e.s.d. in the dihedral angle between two l.s. planes) are estimated using the full covariance matrix. The cell e.s.d.'s are taken into account individually in the estimation of e.s.d.'s in distances, angles and torsion angles; correlations between e.s.d.'s in cell parameters are only used when they are defined by crystal symmetry. An approximate (isotropic) treatment of cell e.s.d.'s is used for estimating e.s.d.'s involving l.s. planes.
Refinement. Refinement of *F*^2^ against ALL reflections. The weighted *R*-factor *wR* and goodness of fit *S* are based on *F*^2^, conventional *R*-factors *R* are based on *F*, with *F* set to zero for negative *F*^2^. The threshold expression of *F*^2^ > σ(*F*^2^) is used only for calculating *R*-factors(gt) *etc*. and is not relevant to the choice of reflections for refinement. *R*-factors based on *F*^2^ are statistically about twice as large as those based on *F*, and *R*- factors based on ALL data will be even larger.

### Fractional atomic coordinates and isotropic or equivalent isotropic displacement parameters (Å^2^)

**Table:**

	*x*	*y*	*z*	*U*_iso_*/*U*_eq_	
Cu1	0.35209 (4)	0.13735 (8)	0.24112 (3)	0.0228 (2)	
O1	0.4485 (2)	0.1048 (4)	0.30394 (14)	0.0225 (7)	
O2	0.4281 (2)	0.4401 (5)	0.31949 (14)	0.0268 (7)	
O3	0.6351 (2)	0.2964 (5)	0.27688 (14)	0.0230 (7)	
O4	0.7598 (2)	0.1491 (5)	0.32317 (15)	0.0274 (7)	
N1	0.6821 (4)	0.1444 (7)	0.5486 (2)	0.0398 (11)	
O5	0.7673 (3)	0.0995 (8)	0.5446 (2)	0.0547 (12)	
O6	0.6397 (3)	0.1487 (6)	0.59882 (18)	0.0476 (11)	
C1	0.4654 (3)	0.2731 (7)	0.33201 (19)	0.0226 (9)	
C2	0.5298 (3)	0.2557 (6)	0.3875 (2)	0.0227 (9)	
C3	0.4867 (4)	0.2677 (6)	0.4447 (2)	0.0266 (10)	
H3	0.4226	0.2964	0.4470	0.032*	
C4	0.5373 (4)	0.2375 (7)	0.4982 (2)	0.0295 (11)	
H4	0.5086	0.2475	0.5366	0.035*	
C5	0.6314 (4)	0.1923 (7)	0.4930 (2)	0.0306 (11)	
C6	0.6779 (4)	0.1873 (7)	0.4368 (2)	0.0279 (10)	
H6A	0.7423	0.1634	0.4350	0.034*	
C7	0.6262 (3)	0.2185 (6)	0.3836 (2)	0.0222 (9)	
C8	0.6772 (3)	0.2218 (6)	0.3224 (2)	0.0221 (9)	
O7	0.2588 (3)	0.1002 (5)	0.30857 (16)	0.0318 (8)	
H7A	0.2064	0.0748	0.2912	0.038*	
H7B	0.2764	0.0051	0.3328	0.038*	
O8	0.4457 (2)	0.2481 (5)	0.18154 (15)	0.0295 (8)	
H8A	0.4672	0.3642	0.1921	0.035*	
H8B	0.4877	0.1567	0.1770	0.035*	

### Atomic displacement parameters (Å^2^)

**Table:**

	*U*^11^	*U*^22^	*U*^33^	*U*^12^	*U*^13^	*U*^23^
Cu1	0.0185 (4)	0.0227 (3)	0.0271 (4)	0.0010 (2)	−0.0029 (2)	−0.00199 (19)
O1	0.0210 (17)	0.0233 (14)	0.0230 (14)	−0.0003 (13)	−0.0020 (13)	−0.0017 (11)
O2	0.0226 (18)	0.0240 (16)	0.0338 (17)	0.0017 (14)	−0.0012 (14)	0.0013 (13)
O3	0.0189 (17)	0.0227 (15)	0.0274 (16)	0.0001 (13)	−0.0008 (13)	0.0015 (12)
O4	0.0188 (18)	0.0333 (17)	0.0301 (17)	0.0047 (14)	0.0015 (14)	0.0047 (13)
N1	0.037 (3)	0.043 (3)	0.039 (3)	−0.006 (2)	0.000 (2)	−0.0008 (18)
O5	0.035 (3)	0.079 (3)	0.050 (3)	0.007 (2)	−0.007 (2)	0.002 (2)
O6	0.052 (3)	0.060 (3)	0.031 (2)	−0.009 (2)	0.0032 (19)	0.0028 (17)
C1	0.018 (2)	0.026 (2)	0.024 (2)	0.0005 (19)	0.0035 (18)	−0.0016 (16)
C2	0.020 (2)	0.0198 (19)	0.028 (2)	−0.0007 (18)	0.0003 (18)	−0.0004 (16)
C3	0.026 (3)	0.024 (2)	0.030 (2)	0.000 (2)	0.001 (2)	−0.0061 (17)
C4	0.034 (3)	0.028 (2)	0.027 (2)	−0.002 (2)	0.001 (2)	−0.0028 (17)
C5	0.041 (3)	0.025 (2)	0.026 (2)	0.000 (2)	−0.007 (2)	−0.0001 (17)
C6	0.023 (3)	0.029 (2)	0.032 (2)	0.002 (2)	−0.001 (2)	−0.0018 (18)
C7	0.022 (2)	0.019 (2)	0.026 (2)	0.0009 (18)	−0.0013 (18)	0.0004 (16)
C8	0.021 (2)	0.018 (2)	0.027 (2)	−0.0036 (18)	−0.0006 (19)	−0.0007 (16)
O7	0.0226 (19)	0.0408 (19)	0.0320 (17)	0.0000 (16)	−0.0039 (15)	−0.0032 (14)
O8	0.0263 (19)	0.0260 (16)	0.0361 (18)	−0.0035 (15)	−0.0035 (15)	0.0024 (13)

### Geometric parameters (Å, °)

**Table:**

Cu1—O4^i^	1.917 (3)	C2—C7	1.393 (7)
Cu1—O1	1.945 (3)	C3—C4	1.382 (7)
Cu1—O8	1.991 (3)	C3—H3	0.9300
Cu1—O7	1.991 (4)	C4—C5	1.373 (7)
Cu1—O3^ii^	2.263 (3)	C4—H4	0.9300
O1—C1	1.278 (5)	C5—C6	1.388 (7)
O2—C1	1.241 (5)	C6—C7	1.385 (7)
O3—C8	1.253 (5)	C6—H6A	0.9300
O4—C8	1.266 (6)	C7—C8	1.514 (6)
N1—O6	1.246 (6)	O7—H7A	0.8505
N1—O5	1.249 (7)	O7—H7B	0.8505
N1—C5	1.441 (7)	O8—H8A	0.8477
C1—C2	1.517 (6)	O8—H8B	0.8487
C2—C3	1.387 (6)		
			
O4^i^—Cu1—O1	175.60 (13)	C4—C3—H3	119.5
O4^i^—Cu1—O8	88.20 (14)	C2—C3—H3	119.5
O1—Cu1—O8	91.45 (14)	C5—C4—C3	117.9 (4)
O4^i^—Cu1—O7	94.90 (15)	C5—C4—H4	121.1
O1—Cu1—O7	86.53 (14)	C3—C4—H4	121.1
O8—Cu1—O7	165.32 (14)	C4—C5—C6	122.8 (5)
O4^i^—Cu1—O3^ii^	88.18 (12)	C4—C5—N1	117.6 (5)
O1—Cu1—O3^ii^	87.58 (12)	C6—C5—N1	119.6 (5)
O8—Cu1—O3^ii^	100.93 (12)	C7—C6—C5	118.6 (5)
O7—Cu1—O3^ii^	93.51 (13)	C7—C6—H6A	120.7
C1—O1—Cu1	112.0 (3)	C5—C6—H6A	120.7
C8—O3—Cu1^iii^	118.6 (3)	C6—C7—C2	119.7 (4)
C8—O4—Cu1^iv^	129.7 (3)	C6—C7—C8	118.8 (4)
O6—N1—O5	122.4 (5)	C2—C7—C8	121.4 (4)
O6—N1—C5	119.2 (5)	O3—C8—O4	126.7 (4)
O5—N1—C5	118.4 (5)	O3—C8—C7	118.0 (4)
O2—C1—O1	124.6 (4)	O4—C8—C7	115.2 (4)
O2—C1—C2	119.8 (4)	Cu1—O7—H7A	106.3
O1—C1—C2	115.4 (4)	Cu1—O7—H7B	110.4
C3—C2—C7	119.9 (4)	H7A—O7—H7B	113.0
C3—C2—C1	116.1 (4)	Cu1—O8—H8A	112.9
C7—C2—C1	123.9 (4)	Cu1—O8—H8B	106.9
C4—C3—C2	121.0 (5)	H8A—O8—H8B	113.8
			
O8—Cu1—O1—C1	81.6 (3)	O5—N1—C5—C6	0.1 (7)
O7—Cu1—O1—C1	−83.9 (3)	C4—C5—C6—C7	−3.5 (7)
O3^ii^—Cu1—O1—C1	−177.6 (3)	N1—C5—C6—C7	175.2 (4)
Cu1—O1—C1—O2	−2.7 (6)	C5—C6—C7—C2	0.5 (7)
Cu1—O1—C1—C2	172.0 (3)	C5—C6—C7—C8	177.5 (4)
O2—C1—C2—C3	70.4 (5)	C3—C2—C7—C6	2.0 (6)
O1—C1—C2—C3	−104.5 (5)	C1—C2—C7—C6	−174.3 (4)
O2—C1—C2—C7	−113.1 (5)	C3—C2—C7—C8	−174.9 (4)
O1—C1—C2—C7	72.0 (6)	C1—C2—C7—C8	8.7 (6)
C7—C2—C3—C4	−1.8 (6)	Cu1^iii^—O3—C8—O4	−90.7 (5)
C1—C2—C3—C4	174.8 (4)	Cu1^iii^—O3—C8—C7	88.5 (4)
C2—C3—C4—C5	−1.0 (7)	Cu1^iv^—O4—C8—O3	3.5 (7)
C3—C4—C5—C6	3.7 (7)	Cu1^iv^—O4—C8—C7	−175.7 (3)
C3—C4—C5—N1	−175.0 (4)	C6—C7—C8—O3	−163.6 (4)
O6—N1—C5—C4	−1.1 (7)	C2—C7—C8—O3	13.3 (6)
O5—N1—C5—C4	178.8 (5)	C6—C7—C8—O4	15.7 (6)
O6—N1—C5—C6	−179.8 (5)	C2—C7—C8—O4	−167.4 (4)

Symmetry codes: (i) *x*−1/2, *y*, −*z*+1/2; (ii) −*x*+1, *y*−1/2, −*z*+1/2; (iii) −*x*+1, *y*+1/2, −*z*+1/2; (iv) *x*+1/2, *y*, −*z*+1/2.

### Hydrogen-bond geometry (Å, °)

**Table:**

*D*—H···*A*	*D*—H	H···*A*	*D*···*A*	*D*—H···*A*
O7—H7A···O2^v^	0.85	2.19	2.862 (5)	136
O7—H7A···O3^i^	0.85	2.30	2.858 (5)	123
O7—H7B···O6^vi^	0.85	2.15	2.959 (6)	158
O8—H8A···O1^iii^	0.85	1.98	2.787 (4)	160
O8—H8B···O2^ii^	0.85	1.85	2.692 (5)	170

Symmetry codes: (v) −*x*+1/2, *y*−1/2, *z*; (i) *x*−1/2, *y*, −*z*+1/2; (vi) −*x*+1, −*y*, −*z*+1; (iii) −*x*+1, *y*+1/2, −*z*+1/2; (ii) −*x*+1, *y*−1/2, −*z*+1/2.
